# Modern Operating Theatres

**Published:** 1898-07-30

**Authors:** 


### MODERN OPERATING THEATRES.

### Dr. John Duncan's Address.

The address delivered by Dr. John Duncan, Consulting Surgeon to the Edinburgh Royal Infirmary, at the opening of the Surgical Section of the British Medical Association was principally devoted to a consideration of the essential requirements of an operating theatre and to a criticism of certain tendencies towards extravagance in the fitting up of modern theatres which have been displayed by some of our hospital managers and hospital architects.

The number of operations performed in hospitals today is so much larger than it used to be in years gone by, that it has become necessary to provide several operating theatres in most large hospitals, and at the Edinburgh Royal Infirmary arrangements have been all but completed whereby a surgical theatre shall be placed at the disposal of each set of wards. Dr. Duncan says that when, not so many years ago, they moved into the present buildings they found one spacious theatre had been provided in which it was intended that all operations should be performed. But now, so wide is the field of surgery and so large is the population from whom patients are drawn, that the youngest of their surgeons performs almost as many operations as were performed in the whole infirmary in the days of Syme.

Moreover, another change has taken place. The operating theatre is not now the centre of attraction that it used to be. Students do not now flock to the surgical theatre as the populace used to flock to an execution—at any rate, they do not do so to the same extent as formerly. The old glorification of mere manual dexterity has greatly diminished; and it is now seen that if one knows what to do there is little difficulty in doing it, "and so a surgical operation has come to be regarded as an incident in a case, and the theatre has become an appanage to the ward, in which whatever manipulation is necessary may be carried out in the way most advantageous to the patient and most beneficial to the student, as a sequel to the diagnosis and a preliminary to the after-treatment."

Speaking of the construction of the theatre, Dr. Duncan says, "I am not of those who have great faith in surgical upholstery. I cannot believe that the expense which has been incurred, here and elsewhere, in transforming cleanly and well-ventilated theatres into structures of tiles and glass was a necessary expenditure. It may be said that all this is in the right direction, but there are considerations worth bearing in mind before the money of a charity is expended in costly reconstruction."

So much has of late been done all over the country, and especially in other countries, to render the walls of operating theatres impervious and washable that Mr. Duncan's remarks on this subject will be read with considerable interest.

"In the first place," he says, "smooth and impermeable materials are not necessarily those which are most easily rendered aseptic. It is a fallacy to suppose that you render a theatre aseptic by douching its floor with antiseptic solutions. I am not sure but that the cleansing of a good old wooden floor is antiseptically as satisfactory as the treatment applied to its modern substitute. In the second place, if the principle be sound on which this change has been made, much more is necessary to carry it to a logical conclusion." No doubt Dr. Duncan is strictly accurate in both these points. The weakness of the first, however, lies in this, that while he speaks of floors, the great change in operating theatres during recent years has been the introduction of washable walls and the removal of all cupboards and receptacles for dust. Modern floors are, no doubt, more easily washed than the old ones, still the old ones were washed, and well washed, too; but the old walls were not. Where the wallss were distempered, which was commonly enough the case, they would be left untouched for a year at a time; and even where they were painted, they certainly could not be washed as frequently, nor as perfectly, without being completely spoiled, as is possible with modern impervious materials. It is the fact that not one part only but the whole of a modern operating-room can be cleansed that is its special feature. The particular material is a detail.

In regard, however, to the second point, we can have no sympathy with Dr. Duncan. He is not the first, nor will he be the last, to trip over the stumbling-block of logical completeness, and we cannot admit that it is any argument against the improvements which have been introduced into modern operating theatres to say that much more is necessary to carry them to their "logical conclusion."

In his search for logical finality Dr. Duncan says: "The emanations from spectators are more dangerous than those from walls and floor. Must we not, therefore, follow those who have cut the spectators off more or less by an impermeable but transparent screen? The emanations from the operator and his assistants are far more likely to reach the wound than those from spectators. So I picture to myself a time when everyone concerned in an operation—patient, surgeons, and assistants—having been rendered from top to toe cutaneously aseptic, shall cover each natural orifice of the body with an antiseptic mask, and, clothing themselves in raiment scientifically pure, shall pass into an atmosphere freed from germs by the air-pump and by heat." But the fact that at present we are far from such a condition of affairs is no reason why we should fold our hands until the day shall arrive when these things shall happen.

It is always useful to hear the views of those who dissent, even when they dissent from progress; but in regard to the construction of operating theatres we think that those who are endeavouring to render them "washable" from top to bottom are on the right tack. Everyone, however, will agree with Dr. Duncan when he says that it would be unfortunate that in cases of antiseptic failure the surgeon should be led to seek in unfavourable surroundings for a loophole of escape from self-blame, or to believe that he cannot operate with perfect success save in a glass case or a crystal palace. A surgeon should be able to operate anywhere; nevertheless, when an institution devotes a room especially and exclusively to operative surgery we think that all the teachings of modern science should be followed in rendering it as well fitted as possible for its purpose,

